# Quantitative Angiography: The Dawn of a New Era in Cardiovascular Medicine

**DOI:** 10.7759/cureus.61407

**Published:** 2024-05-31

**Authors:** Rucha Sawant, Sourya Acharya, Sunil Kumar, Pranav Chaudhari

**Affiliations:** 1 Internal Medicine, Jawaharlal Nehru Medical College, Datta Meghe Institute of Higher Education and Research, Wardha, IND

**Keywords:** interventional cardiology, precision medicine, angiography evolution, diagnostic imaging, cardiovascular medicine, quantitative angiography

## Abstract

This comprehensive review explores the transformative role of quantitative angiography in the landscape of cardiovascular medicine. Tracing the historical evolution of cardiovascular diagnostics, we emphasize the significance of angiography in diagnosis and treatment. The primary focus on quantitative angiography reveals its capacity to move beyond qualitative assessments, providing clinicians with precise measurements and objective parameters. This paradigm shift enhances diagnostic accuracy, promising far-reaching implications for the future of cardiovascular medicine. The ability to tailor interventions based on meticulous measurements optimizes therapeutic strategies and positions the field on the brink of a new era where personalized approaches become the norm. However, challenges such as image quality, radiation exposure, and interpretation variability persist, necessitating a collective call to action for continued research and development. As we confront these issues, collaborative efforts across disciplines are essential to refine existing technologies and usher in innovative solutions. This review concludes with a resounding call for ongoing research initiatives, large-scale clinical studies, and collective commitment to propel quantitative angiography into a universally accepted standard, ensuring its full realization in enhancing patient care and outcomes in cardiovascular medicine.

## Introduction and background

Cardiovascular medicine has witnessed a remarkable evolution over the years, marked by groundbreaking advancements in diagnostic and therapeutic approaches. From the early days of rudimentary techniques to the sophisticated methodologies employed today, the field has progressed substantially, enhancing our understanding of cardiovascular diseases. Central to this evolution is the pivotal role of angiography, a diagnostic imaging technique that has become integral in managing cardiovascular conditions [1]. The journey of cardiovascular medicine dates to pioneering efforts to comprehend the intricacies of the circulatory system. Historical milestones, such as the development of the first catheterization techniques, laid the foundation for further exploration into cardiovascular diagnostics. The continuous refinement of imaging technologies and interventional procedures has propelled the field to unprecedented heights [2]. Angiography, a cornerstone in cardiovascular imaging, has proven indispensable in diagnosing and treating cardiovascular disorders. By providing detailed visualizations of blood vessels and the heart, angiography enables clinicians to pinpoint abnormalities, assess blood flow dynamics, and precisely guide therapeutic interventions. Its role extends beyond mere visualization, serving as a diagnostic compass that aids in formulating effective treatment strategies [3].

This comprehensive review aims to delve into the dynamic landscape of cardiovascular medicine with a specific focus on the transformative role played by quantitative angiography. As we navigate the intricate network of blood vessels and delve into the cardiac chambers, we aim to shed light on the advancements, applications, and implications of quantitative angiography. Quantitative angiography represents a paradigm shift in cardiovascular imaging, transcending traditional qualitative assessments. By introducing precise measurements and quantitative parameters, this technique empowers clinicians to move beyond visual approximations, facilitating a more objective evaluation of vascular anatomy and pathology. The review will illuminate the intricacies of quantitative angiography, emphasizing its significance in enhancing diagnostic accuracy. The integration of quantitative angiography into clinical practice has far-reaching implications for the field of cardiovascular medicine. From refining diagnostic capabilities to guiding therapeutic interventions, the impact is multifaceted. This review will explore and analyze how quantitative angiography improves patient outcomes and personalized treatment strategies and advances our overall understanding of cardiovascular diseases.

## Review

Basics of quantitative angiography

Principles and Fundamentals

Image acquisition and processing: Quantitative coronary angiography (QCA) is a methodology that provides objective numerical data in conjunction with visual assessment. It employs rapid, computerized angiographic analysis systems to quantify and evaluate the extent of coronary artery stenosis. One of the primary stages in the QCA process involves image acquisition and processing. QCA relies on contrast coronary angiography, utilizing swift computerized analysis systems to derive objective and interval measures of coronary artery parameters. These measures are essential for accurate assessment [4,5]. Furthermore, the advancement to 3D QCA has been significant. This method, which necessitates a minimum of two angiographic projections, addresses the limitations of traditional 2D QCA. By offering more precise assessments, 3D QCA holds promise for the future. Moreover, potential advancements may integrate artificial intelligence (AI) and the Internet further to improve decision-making in percutaneous coronary intervention (PCI) [6]. The utility and limitations of QCA are worth noting. While it has found extensive application in clinical research and specific instances of clinical practice, its efficacy depends on image quality. QCA may face challenges when evaluating complex lesions, particularly those with thrombus or calcification. Nonetheless, its reproducibility and validity make it a valuable tool [4,7]. Regarding the analytical process, QCA employs automated edge detection of the contrasted blood vessel. This technique demonstrates superior accuracy and reproducibility compared to visual estimation. Consequently, QCA serves as a valuable means of appraising coronary artery stenosis, offering objective numerical data and overcoming some limitations associated with visual estimation. As the field progresses, future developments may involve integrating advanced technologies and addressing ongoing challenges in assessing complex lesions [4,6,7].

Measurement parameters: QCA yields precise numerical data and visual assessments. This technique relies on contrast coronary angiography and employs swift, computerized analysis systems to obtain parameters that objectively quantify, utilizing interval measures. Commonly utilized variables derived from QCA encompass minimal lumen diameter (MLD), reference vessel diameter, acute lumen gain (final MLD - baseline MLD) following PCI, late lumen loss (follow-up MLD - final MLD) post-PCI, and percent diameter stenosis [8]. QCA measurements encompass the percentage diameter of stenosis and the percentage area of stenosis, demonstrating greater accuracy and reproducibility than visual estimation [9]. Other parameters acquired through QCA include MLD (the smallest diameter of the lumen) and reference diameter [6]. The analytical process in QCA involves the automated edge detection of the contrasted blood vessel [6]. QCA furnishes a spectrum of measurement parameters that provide objective numerical data, addressing certain limitations associated with visual estimation [6,8,9].

Comparison With Traditional Angiography

QCA presents several advantages over traditional visual estimation in evaluating coronary artery stenosis. A comparative study between QCA and visual estimation revealed a statistically significant difference in QCA parameters, precisely the percentage diameter and percentage area of stenosis. This underscores the potential for more precise and reproducible measurements with QCA [9]. Compared to coronary angiography, QCA directly acquires parameters that offer objective quantification with interval measures, thereby overcoming the limitations associated with visual estimation [5]. However, it is crucial to acknowledge notable variations in the performance of currently available QCA systems, as evidenced by a study on the comparative validation of QCA systems [10]. Despite these differences, QCA remains a crucial tool in clinical trials and post-marketing surveillance of coronary artery disease [6]. Consequently, while QCA provides more accurate and reproducible measurements than visual estimation, users must be mindful of the potential variability in the performance of different QCA systems for clinical assessment [6,9,10].

Importance of Accurate Quantitative Assessment in Diagnosis

Quantitative evaluation is pivotal in diagnosing coronary artery disease, significantly influencing decision-making and treatment planning. QCA, a long-established method in clinical research and selectively in clinical practice, has gained prominence due to its reproducibility and validity [7]. QCA stands out by furnishing objective numerical data and has demonstrated superior accuracy and reproducibility compared to visual estimation when assessing coronary artery stenosis [4,7]. This significance is underscored by the revelation that, in numerous instances, QCA identified treated lesions as less severe than perceived through physician visual assessment, thereby emphasizing potential disparities between the two methods [7]. Moreover, despite proven benefits in decision-making, adjunctive functional assessments of lesion severity, such as fractional flow reserve (FFR), are infrequently incorporated into practice, accentuating the pressing need for enhanced precision in quantitative assessment during diagnosis [7]. In essence, the imperative for accurate quantitative assessment in diagnosing coronary artery disease is reinforced by the observed disparities between visual assessment and QCA, coupled with the documented advantages of incorporating adjunctive functional assessments. This emphasizes the necessity for more consistent and precise diagnostic methodologies [7].

Quantitative angiography vs. angioscopy vs. intravenous sonography

Comparative Analysis of Imaging Techniques

Quantitative angiography, angioscopy, and intravascular sonography (IVUS) constitute imaging modalities for diagnosing and treating cardiovascular diseases. QCA stands out as a computerized analysis system for angiography, demonstrating superior accuracy and reproducibility compared to visual estimation in evaluating coronary artery stenosis [11]. Angioscopy facilitates direct visualization of the coronary lumen and plaque morphology, while IVUS generates cross-sectional images of the vessel wall and lumen [12]. Numerous studies have undertaken a comparative analysis of these techniques, revealing, for instance, IVUS's heightened sensitivity in detecting calcium in stable patients compared to angiography [12]. Another study compared three-dimensional QCA (3D-QCA) and IVUS in predicting functionally significant coronary lesions, establishing their comparable diagnostic accuracy [13]. Although QCA has found widespread application in clinical research and practice, it does have limitations, relying on image quality and facing challenges in evaluating complex lesions like those involving thrombus or calcification [7]. Angioscopy and IVUS each have distinctive advantages and limitations, and the choice of technique hinges on the clinical scenario and the specific information sought [11-13]. Numerous studies have conducted comparative analyses of quantitative angiography, angioscopy, and IVUS, affirming that while each method bears its unique strengths and weaknesses, they are invaluable tools in diagnosing and treating cardiovascular diseases. The selection among these techniques is contingent upon the clinical context and the precise information required [11-13].

Strengths and Limitations of Each Modality

QCA is a computerized angiographic analysis system that has demonstrated superiority over visual estimation in accurately assessing coronary artery stenosis [11]. Its notable advantages lie in its objectivity, reproducibility, and relatively modest cost [14]. However, QCA is not without its limitations, as its effectiveness hinges on image quality, and it encounters difficulties in evaluating complex lesions, particularly those involving thrombus or calcification [14]. Angioscopy, another technique in cardiovascular assessment, enables direct visualization of the coronary lumen and plaque morphology [11]. Its benefits include the ability to directly observe vessel and plaque morphology, aiding in diagnosing and treating cardiovascular diseases [11]. Nonetheless, angioscopy requires refinement due to its invasive nature, limited availability, and inability to provide cross-sectional images of the vessel wall and lumen [11]. IVUS provides cross-sectional images of the vessel wall and lumen, facilitating a more precise assessment of plaque morphology and vessel dimensions [12]. IVUS's strengths include its ability to offer detailed information about vessel and plaque morphology, thereby enhancing the diagnosis and treatment of cardiovascular diseases [12]. Despite these advantages, IVUS has limitations, such as invasiveness, higher cost compared to QCA, and restricted availability in certain catheterization laboratories [12]. Each modality-QCA, angioscopy, and IVUS-possesses distinct advantages and limitations. Selecting a technique depends on the clinical scenario and specific information requirements [11,12,14]. QCA emerges as a relatively cost-effective and objective method, while angioscopy allows for direct vessel and plaque morphology visualization. IVUS, offering detailed cross-sectional images of the vessel wall and lumen, is more costly and invasive than QCA [11,12,14].

Clinical Scenarios Favoring One Technique Over Another

QCA is a computerized angiographic analysis system renowned for providing precise and replicable evaluations of coronary artery stenosis [11]. Distinguished by its rapid assessment capabilities, QCA finds widespread application in clinical research and, in certain instances, clinical practice, owing to its reproducibility and validity [15]. Angioscopy enables direct visualization of the luminal surface of blood vessels, facilitating the assessment of plaque characteristics and thrombus burden [11]. This modality offers a distinct advantage in scrutinizing plaque features and thrombus burden, aspects that may not be fully captured by alternative imaging techniques [11]. Intravascular ultrasound (IVUS) produces cross-sectional images of blood vessels, allowing for an accurate assessment of plaque burden, vessel size, and stent optimization [14]. IVUS is preferred in scenarios requiring detailed insights into plaque burden, vessel size, and stent optimization, particularly beneficial in complex lesions, such as those with extensive calcification, where 2D angiography may have limitations [12]. The selection of a technique depends on the specific clinical scenario. QCA excels in swiftly evaluating coronary artery stenosis, while angioscopy and IVUS are favored for exploring plaque characteristics, thrombus burden, and comprehensive vessel and lesion morphology. Each modality offers unique advantages, selected based on the specific demands of the clinical situation.

Clinical applications

Coronary Artery Disease

Quantitative assessment for stenosis severity: QCA proves to be a valuable instrument for evaluating coronary artery stenosis in clinical settings. It offers an objective and reproducible assessment of lumen diameter, a critical factor for procedures like PCI [6]. QCA has demonstrated superior accuracy and reproducibility compared to visual estimation in appraising coronary artery stenosis, establishing itself as a widely utilized method in clinical research and, in specific instances, clinical practice [4,6]. In the clinical realm of coronary artery disease, QCA assumes a pivotal role by furnishing precise and objective measurements of stenosis severity. A consensus statement has underscored its significance as the standard for stenosis assessment, imparting valuable insights for treatment and procedural planning [16]. Furthermore, QCA has been incorporated into prospective, randomized, double-blind, and placebo-controlled investigations to quantitatively gauge the entire coronary artery system, showcasing its clinical relevance in evaluating the progression of coronary artery disease [17]. QCA is a crucial tool for quantitatively assessing stenosis severity in the context of coronary artery disease, providing objective and reproducible measurements essential for clinical decision-making and research endeavors.

Evaluation of coronary flow reserve: The assessment is crucial in evaluating coronary artery disease and devising appropriate treatment strategies. Two commonly utilized methods are QCA and FFR. QCA is a computerized angiographic analysis system designed to provide precise and reproducible evaluations of coronary artery stenosis [17]. However, it primarily focuses on assessing the severity of stenosis rather than directly evaluating coronary flow reserve. In contrast, FFR measures the blood flow ratio through a stenotic coronary artery to the normal flow in the same artery during rest and exercise [18]. Recognized as the gold standard for evaluating coronary flow reserve, FFR requires a pressure wire, which may present challenges for certain patients due to contraindications or logistical reasons. A comparative study assessing the diagnostic performance of angiography (including visual estimation and QCA) versus FFR revealed that FFR demonstrated a sensitivity of 76% and specificity of 76% according to random summary receiver-operator characteristic estimates [19]. Conversely, another study comparing FFR with QCA aimed to evaluate the diagnostic performance of angiography conducted through visual estimation and QCA against FFR [20]. While QCA remains a valuable tool for assessing coronary artery stenosis, FFR is the preferred method for evaluating coronary flow reserve. However, the choice between these methods depends on the specific clinical scenario and the availability of resources.

Peripheral Artery Disease

Quantitative angiography in assessing peripheral vessels: Quantitative angiography is a crucial instrument in assessing peripheral artery disease (PAD), aiding in determining disease severity. Numerous studies have delved into applying quantitative angiography for evaluating peripheral vessels, revealing its significance in this domain. In one study, an exploration of intra- and interobserver variability in the visual and computerized assessment of peripheral arterial disease demonstrated that computerized assessment outperformed visual estimation regarding accuracy and reproducibility [21]. Additionally, another study focused on the direct quantitative assessment of collateral circulation in peripheral arteries during angiography, revealing that quantitatively assessed collateral arterial function at rest proved sufficient to prevent ischemia [22]. Beyond quantitative angiography, alternative imaging modalities such as contrast-enhanced magnetic resonance angiography (MRA) and peri-interventional fluoroscopic imaging have been employed for the quantitative evaluation of peripheral arterial blood flow [23,24]. These investigations have showcased these methods' feasibility and clinical utility in the comprehensive assessment of PAD. Quantitative angiography is an invaluable tool for evaluating PAD and gauging disease severity. The superiority of computerized assessment over visual estimation and the successful utilization of other imaging modalities like MRA and fluoroscopic imaging underscores the multifaceted approach to quantitatively assessing peripheral arterial health.

Role in endovascular interventions: Endovascular intervention is a valuable treatment modality for PAD, providing a less risky alternative to open surgery, particularly for patients grappling with multiple comorbidities [25]. Balloon angioplasty and stenting constitute the cornerstone of endovascular therapy for PAD, with recent advancements introducing novel elements like drug-eluting stents and drug-coated balloons [25]. Additional supplementary devices, such as atherectomy, play specific roles in navigating chronic total occlusions or reducing plaque burden [25]. Recent progress in endovascular treatment has expanded the options available for managing PAD, presenting numerous alternatives for patients whose conditions are amenable to endovascular therapy [26]. The Trans-Atlantic Inter-Society Consensus Document on the Management of Peripheral Arterial Disease advocates for balloon angioplasty and stenting in cases involving stenosis of the aortoiliac segments [27]. Nonetheless, uncertainty persists regarding the most efficacious treatment strategy for PAD patients, and the selection of treatment hinges on the particulars of the clinical scenario. Endovascular intervention, as a valuable treatment avenue for PAD, offers a lower-risk alternative to open surgery for a considerable number of patients. Balloon angioplasty and stenting serve as the foundational components of endovascular therapy, while ongoing research is imperative to ascertain the most effective treatment strategy for individuals with PAD.

Structural Heart Interventions

Quantitative assessment in transcatheter aortic valve replacement: Quantitative assessment is pivotal in transcatheter aortic valve replacement (TAVR), particularly in evaluating acute regurgitation post-procedure. Numerous studies have concentrated on quantifying regurgitation following TAVR, underscoring its significance in the post-procedural assessment. A multicenter pooled analysis of 2258 valves aimed to appraise acute regurgitation after TAVR, comparing various implanted transcatheter heart valves [28]. This study underscored the importance of quantitative assessment in scrutinizing the outcomes of TAVR procedures. Another study, the OVAL study, demonstrated the simplicity, reproducibility, and validity of the quantitative aortographic assessment of aortic regurgitation after TAVR using video densitometry [29]. These findings emphasize the value of quantitative assessment techniques in the post-procedural evaluation of TAVR. Furthermore, computed tomography (CT) has been designated a valuable tool for diagnosing structural heart interventions, including TAVR. CT plays a crucial role in patient selection, pre-procedural planning, device sizing, and post-procedural assessment, highlighting the comprehensive use of quantitative imaging in the context of TAVR and other structural heart interventions [30]. Using quantitative assessment techniques, such as aortographic assessment and CT imaging, is integral in evaluating TAVR outcomes, particularly in assessing acute regurgitation and ensuring the procedure's effectiveness. These studies demonstrate the ongoing focus on advancing quantitative assessment methods to enhance the quality and precision of TAVR procedures.

Application in other structural heart interventions: TAVR has become a cornerstone in structural heart interventions, where quantitative assessment plays a crucial role in evaluating outcomes, particularly in assessing acute regurgitation post-procedure [31]. This necessity for quantitative assessment extends beyond TAVR to encompass other structural heart interventions, such as percutaneous interventions in adult congenital heart disease, providing invaluable insights for comprehensive procedure evaluation [31]. In the realm of imaging modalities, patient-specific modeling, which includes techniques like 3D printing and CT, is indispensable for pre-procedural planning, device sizing, and post-procedural assessment across various structural heart interventions, highlighting the importance of quantitative imaging techniques [30,32]. CT is pivotal in preoperative evaluation and planning, especially for patients undergoing transcatheter interventions for structural heart diseases like TAVR and mitral valve replacement [33]. The ongoing evolution and expanding indications in structural heart interventions underscore the increasing importance of quantitative assessment in ensuring the effectiveness and precision of these procedures [31]. Quantitative assessment techniques are integral to various structural heart interventions, including TAVR, and are crucial for pre-procedural planning, intra-procedural evaluation, and post-procedural outcomes assessment. Integrating advanced imaging modalities and patient-specific modeling further enhances these interventions' precision and clinical impact.

Challenges and limitations

Image Quality and Resolution Challenges in QCA

QCA encounters limitations and challenges related to image quality, particularly when assessing complex lesions with features such as thrombus or calcification [7]. The accuracy of QCA is intricately tied to the quality and resolution of the angiographic images [8]. Subjectivity in analysis poses a significant challenge, especially in multi-case research designs [34]. Offline QCA, however, enables more precise comparisons of changes in vessel dimensions and the identification of endothelial dysfunction [8]. The future trajectory of QCA may witness advancements through the integration of AI and the Internet, offering potential improvements in decision-making for PCI [6].

Concerns Regarding Radiation Exposure

Exposure to radiation, including medical radiation, carries potential health risks, with the probability of adverse effects directly proportional to the received dose. While no level of radiation exposure is entirely risk-free, principles of radiation protection, including justification, optimization, and dose limitation, aim to minimize associated risks [35,36]. In medical procedures like QCA, it is crucial to balance the benefits and risks, minimize exposure duration, maintain safe distances, and employ physical shielding to safeguard patients and healthcare workers [37]. Acknowledging the value of medical radiation in diagnosis and treatment, it is imperative to adhere to established principles and protective measures in medical settings.

Interpretation Variability in QCA

QCA grapples with interpretation variability from inconsistencies in image acquisition, frame selection, vessel tone variations between measurements, and differences in interpretation across observers and laboratories [38,39]. Studies comparing clinical interpretation with visual assessment and QCA reveal hospital-based variations in mean percent diameter stenosis and confidence intervals [40]. Standardization efforts in QCA protocols and establishing core laboratories for centralized analysis aim to mitigate this challenge [41]. Moreover, integrating advanced technologies like AI holds promise for reducing interpretation variability in the future [39].

Technological Challenges and Advancements in QCA

**Figure 1:**
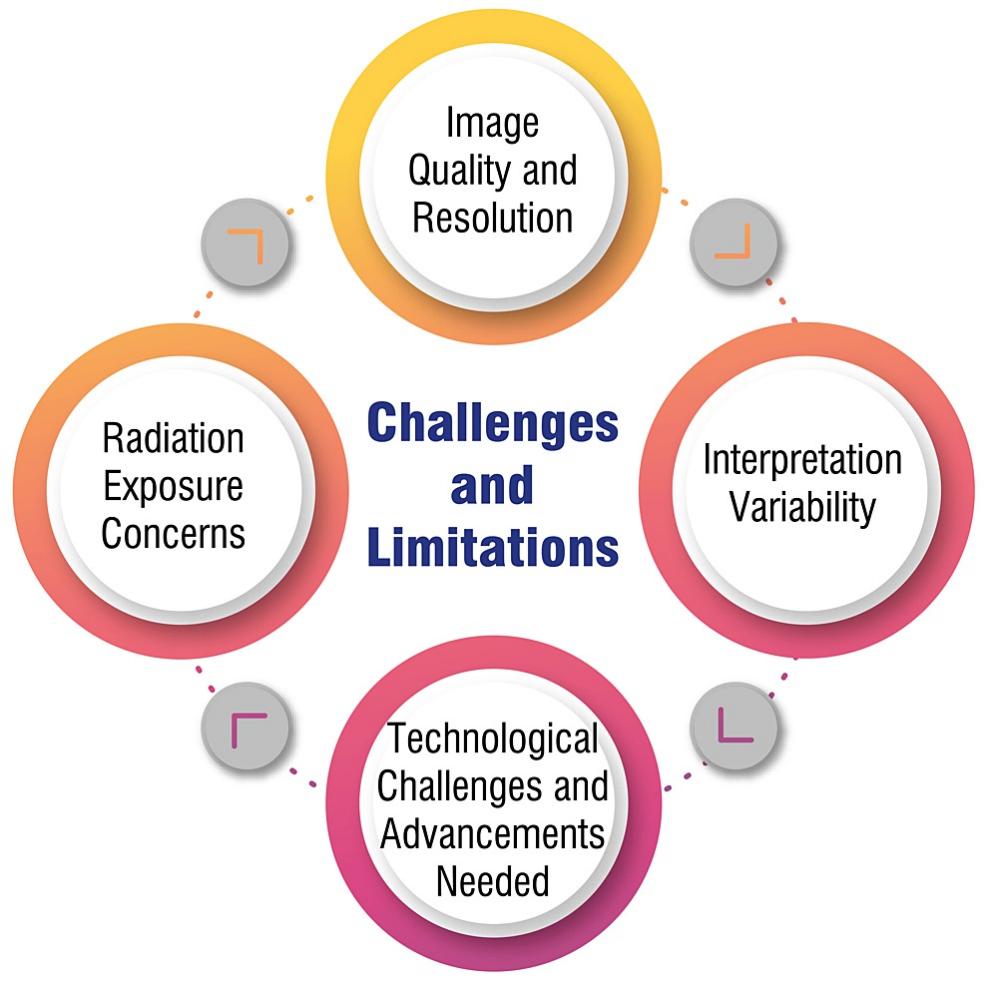
Challenges and limitations Image Credit: Dr. Rucha Sawant

Addressing challenges in QCA necessitates technological advancements, particularly in interpretation variability related to image acquisition and analysis differences between observers and laboratories [42-44]. Standardization efforts and core laboratory establishments have been initiated to enhance consistency [34,44]. The incorporation of AI into QCA (AI-QCA) shows promise, as recent studies demonstrate its accuracy and consistency comparable to intravascular ultrasound (IVUS) in assessing coronary artery stenosis [44]. AI-QCA holds the potential to instill confidence in treating physicians and improve clinical decision-making, although further research is warranted to explore its clinical utility and safety fully [44]. Challenges and limitations are shown in Figure 1.

Future directions

Integration of AI in Quantitative Angiography

AI in QCA holds significant promise for addressing current challenges and advancing cardiovascular care. AI-based QCA demonstrates the potential for automated and real-time analysis of coronary angiography images, providing accurate and consistent measurements of coronary stenotic lesions comparable to intravascular ultrasound (IVUS) [44,45]. This technological advancement has implications for improved treatment guidance, risk stratification, and enhanced patient outcomes [46]. However, challenges, such as addressing AI bias and ensuring external validation of diverse patient populations, must be overcome for successful development and implementation [46]. Rigorous research is essential to fully explore the clinical utility and safety of AI-based QCA in real-world clinical practice [44,45]. Despite these challenges, recognizing AI technology's potential in analyzing coronary angiography marks a remarkable advancement in cardiovascular care [42], requiring ongoing research and development to ensure accuracy, reliability, and safe integration.

Advancements in Imaging Technologies

Advances in imaging technologies hold great promise for the future of QCA. The integration of AI and machine learning (ML) in medical imaging, including QCA, offers automated and real-time analysis comparable to intravascular ultrasound (IVUS) [42,47,48]. Beyond AI, other innovations, such as augmented reality, virtual reality, and 3D medical imaging, have transformative implications in healthcare [49]. Additionally, cloud-based solutions, workflow optimization, and cyber resiliency strategies are shaping the landscape of enterprise imaging, including QCA [50]. These advancements aim to enhance the accuracy, reliability, and safety of QCA, leading to improved treatment guidance, risk stratification, and optimal patient outcomes [42,47,50,51]. However, further research and development are imperative to ensure these technologies' accurate, reliable, and safe integration into clinical practice [42,47,50,51].

Potential Impact on Personalized Medicine

The integration of AI and ML into medical imaging, including QCA, holds substantial potential for impacting personalized medicine [52-54]. AI-based QCA, providing automated and real-time analysis of coronary angiography images, offers accurate and consistent measurements comparable to intravascular ultrasound (IVUS) [52,53]. This has implications for improved treatment guidance, risk stratification, and optimal patient outcomes [54]. Precision medicine, a key component of personalized medicine, is further empowered by data collection and analysis techniques, including genomics, social/behavioral determinants, and environmental knowledge [52]. These techniques precisely characterize health and disease states, guiding tailored therapeutic options [52]. The integration of AI in QCA and the utilization of precision medicine techniques have transformative potential in providing personalized and precise medical treatment tailored to individual characteristics.

Emerging Trends and Research Directions

Current trends and research directions in QCA encompass the integration of AI and ML in medical imaging, showcasing the potential for automated real-time analysis comparable to intravascular ultrasound (IVUS) [50-54]. Advancements in imaging technologies, including augmented reality, virtual reality, and 3D medical imaging, are gaining traction within the healthcare industry [54]. Furthermore, cloud-based solutions, workflow optimization, and cyber resiliency strategies are shaping the trajectory of enterprise imaging, including QCA [50]. These technologies can enhance accuracy, reliability, and safety, improving treatment guidance, risk stratification, and optimal patient outcomes [50-54]. Continued research and development are imperative to ensure these technologies' accurate, reliable, and safe integration into clinical practice [50-54].

## Conclusions

In conclusion, this review's comprehensive exploration of quantitative angiography illuminates its transformative impact on cardiovascular medicine. Through a historical lens, we traced the evolution of cardiovascular medicine and recognized the pivotal role of angiography in diagnosis and treatment. The central focus on quantitative angiography reveals its capacity to transcend traditional qualitative assessments, providing clinicians with precise measurements and objective parameters. This shift toward quantitative precision enhances diagnostic accuracy and, consequently, holds promising implications for the future of cardiovascular medicine. Tailoring interventions based on meticulous measurements optimizes therapeutic strategies and positions the field on the cusp of a new era where personalized approaches become the norm. However, challenges persist, demanding a collective call to action for continued research and development. As we confront issues like image quality, radiation exposure, and interpretation variability, collaborative efforts across disciplines are essential to refine existing technologies and usher in innovative solutions. This review, therefore, concludes with a resounding call for ongoing research initiatives, large-scale clinical studies, and a collective commitment to propel quantitative angiography from a promising technique to a universally accepted standard in cardiovascular imaging, ensuring its full realization in enhancing patient care and outcomes.
